# A scoping review of the potential for chart stimulated recall as a clinical research method

**DOI:** 10.1186/s12913-017-2539-y

**Published:** 2017-08-22

**Authors:** Carol Sinnott, Martina A. Kelly, Colin P. Bradley

**Affiliations:** 1 Department of General Practice, University College Cork, Cork, Ireland; 2 Cambridge Centre for Health Services Research, Department of Public Health & Primary Care, University of Cambridge, Cambridge, UK; 3 Department of Family Medicine, University of Calgary, Alberta, Canada

**Keywords:** Chart stimulated recall, Scoping review, Qualitative research, General practice, Decision making

## Abstract

**Background:**

Chart-stimulated recall (CSR) is a case-based interviewing technique, which is used in the assessment of clinical decision-making in medical education and professional certification. Increasingly, clinical decision-making is a concern for clinical research in primary care. In this study, we review the prior application and utility of CSR as a technique for research interviews in primary care.

**Methods:**

Following Arksey & O’Malley’s method for scoping reviews, we searched seven databases, grey literature, reference lists, and contacted experts in the field. We excluded studies on medical education or competence assessment. Retrieved citations were screened by one reviewer and full texts were ordered for all potentially relevant abstracts. Two researchers independently reviewed full texts and performed data extraction and quality appraisal if inclusion criteria were met. Data were collated and summarised using a published framework on the reporting of qualitative interview techniques, which was chosen a priori. The preferred reporting items for systematic reviews and meta-analyses (PRISMA) guidelines informed the review report.

**Results:**

From an initial list of 789 citations, eight studies using CSR in research interviews were included in the review: six from North America, one from the Netherlands, and one from Ireland. The most common purpose of included studies was to examine the influence of guidelines on physicians’ decisions. The number of interviewees ranged from seven to twenty nine, while the number of charts discussed per interview ranged from one to twelve. CSR gave insights into physicians’ reasoning for actions taken or not taken; the unrecorded social and clinical influences on decisions; and discrepancies between physicians’ real and perceived practice. Ethical concerns and the training and influence of the researcher were poorly discussed in most of the studies. Potential pitfalls included the risk of recall, selection and observation biases.

**Conclusions:**

Despite the proven validity, reliability and acceptability of CSR in assessment interviews in medical education, its use in clinical research is limited. Application of CSR in qualitative research brings interview data closer to the reality of practice. Although further development of the approach is required, we recommend a role for CSR in research interviews on decision-making in clinical practice.

**Electronic supplementary material:**

The online version of this article (doi:10.1186/s12913-017-2539-y) contains supplementary material, which is available to authorized users.

## Background

Since the early 1980s, chart stimulated recall (CSR) has been used to assess the competency of practicing physicians [1]. Originally described for the re-certification of emergency physicians, CSR is a case-based interviewing technique used to examine clinical decision-making [2]. Chart-based medical notes are sometimes short or incomplete and reviewing the chart alone may not offer sufficient information to understand why or how clinical decisions were made. The premise of chart stimulated recall is that asking physicians to describe a clinical encounter using the patient’s chart to prompt their recollection of events will lead to a narrative that has greater detail than the medical notes or the participants account alone and allows an interviewer to probe the reasons why certain decisions were made. Empirical research supports the acceptability, reliability, and validity of CSR in competence assessment, and shows that it allows a more thorough assessment of clinical practice than chart review alone [3, 4]. More recently, CSR has been adopted as a means of assessing decision-making in postgraduate medical education [5]. In this setting, it has been found to be useful for delivering immediate feedback on specific patient encounters to residents, enhancing their understanding of the competencies being evaluated, and encouraging reflective practice [6].

Given the proven value of CSR in generating information on clinical decision-making in regulatory and educational settings, it would seem a useful technique in clinical research interviews in primary care. Clinical decision-making is a complex process which incorporates both slow deliberate and fast intuitive cognitions [7]. It requires the synthesis of conscious and sub-conscious information on the signs and symptoms of disease, patients’ values, treatment options, and available healthcare resources [8]. In primary care, research on clinical decision-making presents additional challenges as many issues may be dealt with in a single clinical encounter [9], record keeping is often brief or incomplete [10], and the diagnosis or course of action is not always clear [11]. Research methods capable of capturing these multiple dimensions are required. However, even in the context of increasing interest in decision-making and complexity in primary care [12], CSR has been infrequently used in clinical research to date.

Recently, we used CSR during qualitative interviews to explore how general practitioners (GPs) make decisions for patients with multiple long-term conditions in primary care [13]. We found the technique was acceptable to GPs, and was an efficient means of generating rich data on the complexities of their clinical practice. Given our positive experience, the aim of this paper was to review the broader application of CSR as a clinical research tool in primary care, to provide an overview for other researchers who may consider using the CSR technique.

## Methods

We conducted a scoping review of the literature using CSR in clinical research in primary care. Scoping reviews are designed to describe relevant literature in a particular area, in contrast to systematic reviews, which seek to answer specific research questions. Scoping reviews are useful for topics that have not been previously reviewed and where many different study designs might be applicable. We followed the five step framework for scoping reviews described by Arksey and O’Malley [14], but incorporated recent refinements to the approach by Levac et al. [15].

### Step 1: Identify the research question

To describe the use of CSR in clinical research studies in primary care, we defined clinical research as research relating to the study and practice of medicine in relation to the care of patients [16]. We excluded studies using CSR for medical education research, which we defined as any investigation relating to the education of medical professionals including curriculum development, teaching, evaluation, research methodology, and use of technology in education [17]. We also excluded studies using CSR for competence assessment or physician licensing purposes, and study protocols where CSR had not yet been used. Based on the assumption that in general practice consultations and charts differ in content from those in secondary care, we restricted the review to studies that were conducted in primary care or included a majority of general practitioners (GPs) or their equivalent [18].

### Step 2: Identify relevant studies

The search was conducted in May 2015, using seven databases on the EBSCO platform: CINAHL, Academic Search Complete, MEDLINE, Psychology and Behavioral Sciences Collection, PsycINFO, Social Sciences Full Text (H.W. Wilson), and SocINDEX. These databases represent a broad scope of academic fields where health related research is published. We used broad search terms which related to general practice and chart stimulated recall (see Additional file 1), and looked for these in any aspect of the title, abstract or paper text. The search was not limited by language, dates of publication or study type. We supplemented this by searching databases of grey literature (WorldCat, Ebooks, Proceedings, and Papersfirst from the OCLC FirstSearch platform), citation and reference lists and contacting experts in the field.

### Step 3: Study selection

The titles and abstracts of all retrieved citations were read by one reviewer (CS). Full texts were ordered for all potentially relevant abstracts. Each full text was reviewed by two researchers (CS, MK) and included if inclusion criteria were met. Exclusion criteria were ranked a priori and once one exclusion criterion had been met, others were not sought. Reasons for exclusion of full texts were recorded and compared between the two reviewers for consistency. Disagreements between reviewers were resolved by consensus discussion of the full text papers, or referral to a third reviewer (CB) where necessary.

### Step 4: Charting the data

All reviewers independently read each included study, extracted relevant data and entered it into a data extraction form (see Additional file 2). Extracted data included: the study aims, setting, participants, means of chart selection, approach to data analysis, and contribution of CSR to the study findings.

### Step 5: Collating, summarising, and reporting the results

This step had three components: analysing data, reporting results and applying meaning to the results including the implications of our findings in a broader context [15]. The data extraction forms for each study were combined and discussed by all authors. The Kendall and Murray framework for describing approaches to qualitative interviews (four specific areas are shown in Table 1) was adapted to structure the analysis and reporting of results [19, 20]. Although quality appraisal is not an original feature of scoping reviews, it was felt that quality appraisal would reveal particular strengths and weaknesses of the CSR literature, and would be important to guide future researchers in the use of the technique [15]. Thus, two reviewers (CS, MK) assessed included studies with the relevant critical appraisal skills programme (CASP) tool [21]. THE PRISMA guidelines [22] informed the review report (see Additional file 3).

**Table 1:** Kendall And Murray framework for describing approaches to qualitative interviewing [19, 20]

Kendall and Murray framework for describing approaches to qualitative interviews [19, 20]	
1.	When is this approach appropriate?
2.	How to use this approach
3.	What type of findings to expect
4.	Potential pitfalls and how to avoid them

## Results

**Fig. 1:**
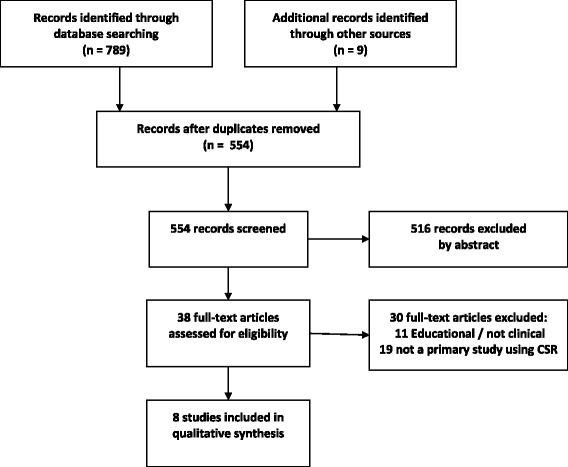
PRISMA flow diagram of literature search

From an initial list of 789 citations, eight studies were included in the review: six from North America, one from the Netherlands, and one from Ireland. Figure 1 shows the PRISMA flow diagram for retrieved citations [22]. The characteristics of the included papers are shown in Tables 2 and 3.

**Table 2:** Characteristics Of Included Studies: Aims, Participants And Setting

First author	Country and Year of Publication	Aims of study	Participants and setting
Ab [25]	Netherlands, 2009	To explore why general practitioners do not follow guidelines on lipid-lowering treatment in patients with type 2 diabetes mellitus, to gain insight into the factors that represent appropriate vs. inappropriate care and tailor interventions to reduce inappropriate care.	7 purposively sampled general practitioners who had indicated that they were familiar with recently distributed guidelines on statin use in diabetic patients.
Dee [27]	USA, 1993	To describe the information needs and information-seeking behaviour of rural physicians with or without hospital library access	12 rural physicians (9 family physicians and 3 specialists) in Central Florida who agreed to participate after an extensive recruitment drive
Guerra [24]	USA, 2007	To assess whether primary care physicians routinely discuss prostate cancer screening and explore the barriers to and facilitators of these discussions	18 purposively sampled, community- and academic-based primary care physicians
Guerra [23]	USA, 2007	To explore the barriers of and facilitators to physician recommendation of colorectal cancer screening	29 purposively sampled, community- and academic-based primary care physicians
Jennett [29]	Canada, 1995	To demonstrate how patients’ charts can be used to provide insights into diagnostic, investigative, and treatment decisions in NSAID gastropathy, to assist in understanding the complexity behind clinical choices	20 family physicians in active, full-time practice in the Calgary city area
Lockyer [28]	Canada, 1991	To obtain information about physician awareness and acceptance of guidelines, determine what influences physicians’ decisions to investigate and treat neonatal hyperbilirubinemia, and identify how physicians preferred to learn about guidelines	25 physicians (5 paediatricians and 20 family physicians) who prescribed phototherapy for jaundiced neonates in the Foothills Provincial Hospital, Calgary.
Rochefort [26]	Canada, 2012	To describe physicians’ decision-making processes, and factors influencing their decisions, regarding treatment choices for hypertension, in order to improve the cost-effectiveness of hypertension management.	29 primary care physicians in Quebec who used a specific clinical information system
Sinnott [13]	Ireland, 2015	To explore how general practitioners make decisions when prescribing for multimorbid patients, with a view to informing intervention design	20 purposively sampled general practitioners in full-time practice in Ireland.

**Table 3:** Characteristics of included studies: charts, interviews and interviewers

First author	How charts were chosen	Topic guide	Average no. of charts per interview	Interview duration	Data analysis	Interviewer (s) background
Ab [25]	Prior to each interview, a list of patients with type 2 diabetes not being prescribed lipid-lowering medication was extracted from the GP’s electronic medical records by the research team.	Open questions on patient, physicians and organisational barriers	10-27 charts: as many as possible were discussed in an hour	60mins	Qualitative: Content analysis	Researcher with unspecified background
Dee [27]	The charts of all patients seen by the physician during half a day of office practice.	Not provided	12 charts	Not provided	Descriptive (mostly quantitative)	Doctoral researcher in librarian studies
Guerra [24]	Interviewee asked to pull 10 charts on men >45 yrs. seen in last 2 weeks, without knowing focus of the study	Unstructured probes informed by the Walsh and McPhee Systems Model of Clinical Preventative Care	2.3 charts	30–45 min	Qualitative: Grounded theory techniques	Medical student
Guerra [23]	Interviewee asked to pull 10 charts on patients >51 years seen in last week, without knowing focus of the study		4.3 charts	30–45 min	Qualitative: Grounded theory techniques	Medical student and physician
Jennett [29]	Standardised patient visit, with chart then used to stimulate recall	Standardised protocol on the rationale for clinical choices, conditions ruled out.	1 chart	20 min	Qualitative: Content analysis	Nurse
Lockyer [28]	The first neonatal case that participating physicians prescribed phototherapy for during the study period.	Closed and open questions on awareness and acceptance of guidelines, and preferred information sources	1 chart	10–15 min	Descriptive (mostly quantitative)	Neonatal nurse
Rochefort [26]	2 cases of hypertension newly started on antihypertensive therapy (one in accordance with guidelines and one not) were purposely selected from the interviewee’s electronic health record database by research team	Literature informed questions on the general approach to hypertension and rationale in chosen cases	2 charts	Not provided	Qualitative: Content analysis	“Trained interviewer”
Sinnott [13]	Interviewee asked to pull 3-5 charts on patients with multiple long-term conditions and 5 + medications, seen the day of or day preceding the interview	Literature informed prompts on management of multimorbidity in primary care	2.5 charts	40–50 min	Grounded theory with constant comparison	General practitioner

### Quality appraisal

Analysing data using the chosen framework and assessing study quality worked synergistically to illuminate the studies. An overview of the CASP quality assessment is available in Additional file 4. In summary, quality appraisal showed that research questions were well aligned with the approach used in seven out of the eight studies. Sufficient descriptions of the qualitative methods used were provided in the five most recent studies [13, 23–26]. The two earliest studies [27, 28] used mainly descriptive statistics to report the results of their analysis.

The influence of the researcher was generally under-reported or not reported, with little reflection by researchers on their own role in the process of data collection. Only one study (13) reflected on the risk of medical interviewers introducing professional biases into the interview, and the measures taken to reduce this risk (i.e. involvement of non-clinical data coders). Most studies, especially the more recent ones, reported ethical approval but few discussed ethical concerns specific to CSR such as confidentiality of patient data, witnessing poor clinical performance, or distressing participants with the CSR discussion.

### When is CSR appropriate?

The definition of CSR and justification for its use varied across studies. Jennett et al. [29] described it as “using the patient’s chart as a stimulus for recall, (while) the physicians were interviewed”. Guerra et al. [23, 24] focused on decision-making, defining CSR as “a physician uses their own documentation of actual patient encounters to stimulate recall of his or her decision-making processes...as an evaluator probes the reasoning behind their medical decision-making”, a definition referenced by two other studies [13, 26]. Lockyer et al. [28] described allowing access to patients’ charts “to allow the physician to elaborate on the process of care and decision-making”. The remaining two studies did not use the term CSR at all: one described interviews where “the GP was able to access the medical record to identify the coded patient” [25], while in the other study “charts of patients seen by the physician following half a day of office practice were reviewed and discussed with the physician” [27].

The most common reason for using CSR was to examine the relationship between clinical guidelines and decision-making. The guidelines under study related to cancer screening [23, 24], or the management of acute or chronic disease [25, 26, 28]. While some studies sought the barriers and facilitators to guideline adherence [23, 24, 28], others focused on the physician characteristics associated with guideline adherence [25, 26], all with the over-arching goal of improving guideline implementation. In two studies, the complexities of clinical care [13, 29] were explored. One study used CSR to reveal the information needs arising for physicians in routine practice [27].

### How to conduct CSR

#### Recruitment

Interviewees were generally recruited by inviting a purposive sample from all eligible participants (Table 2) [13, 23–26, 29], but one study prospectively recruited all physicians who had prescribed the same treatment (viz phototherapy for neonates in a special care baby unit) during the study period [28]. Recruitment was often challenging. Dee et al. [27] undertook an “extensive search…took considerable effort, patience and accommodation” to recruit twelve participants. Of those invited to participate, only 6% agreed in Jennett et al. [29], 19% in Guerra et al. [24], and 36% in Rochefort et al. [26]. The second study by Guerra et al. [23] fared better with 50% acceptance yet the authors still acknowledged this as a study limitation. Aside from lack of time or interest, the reasons for low recruitment were not discussed. Small financial incentives were offered to participants in both studies by Guerra et al.

#### Choosing the charts

Numerous approaches were used to select charts for discussion (see Table 3). For example, in Lockyer et al. [28], each participant’s first incident case of phototherapy during the study period was chosen. In Rochefort et al. [26] the researchers selected two charts: one patient who was treated according to hypertensive guidelines and one who was not. Other studies used larger numbers of charts: Dee et al. [27] discussed all patients seen in the preceding half day of practice, while Ab et al. [25] attempted to discuss all patients who were not treated according to the lipid-lowering guidelines if time permitted.

Jennett et al. [29] used the chart of a standardised patient. Although the GP was aware that a standardised patient would present to their practice, they were unaware of the patient’s identity or their presenting condition. The patient attended the GP for a normal consultation and after the consultation, the chart was used in a qualitative interview to stimulate the physician’s recall of their management.

In both studies by Guerra et al. [23, 24] interviewees were asked to select patients of a particular age and gender seen within a defined period of time, without knowledge of the research question. In one study [13], interviewees were asked to choose patients with multiple long-term conditions who were prescribed five or more medications and had been seen on the day of or day preceding interview [13]. This allowed discussion of a range of disease combinations and issues relating to polypharmacy.

The average number of charts discussed and the duration of interviews is shown in Table 3. Guerra et al. [23, 24] based their assertion that three to five charts were sufficient to assess decision-making on the competence assessment literature. No study offered any other empirical evidence for the number of charts used.

#### Topic guide

Most studies described two phases to the CSR interviews. The opening phase involved general questions. These questions were based on literature reviews [13, 26], the results of a recent local audit [28], or theoretical models [23, 24]. The second phase involved discussion of the chosen patient charts. Here, topic guides ranged from open-ended prompts [13, 23–26] to highly structured closed questions [28, 29]. For example, in Sinnott et al. [13] interviewees were prompted to describe the management of each patient in a chronological, narrative fashion. In contrast, two studies used structured closed questions with multiple choice answers [28, 29]. The latter studies were conducted when CSR was emerging as a method of competence assessment and explicit assessment criteria were necessary, which may explain their more structured approach. None of the published papers discussed how the topic guide may have influenced the content of the case discussion.

#### Analysis

Grounded theory [13, 23, 24] and inductive content analysis [26, 29] were the most commonly used approaches to qualitative data analysis. Authors also combined different qualitative methods, such as using content analysis with constant comparison [25].

#### Triangulation

Triangulation of CSR findings was performed in four studies. In the study on prostate cancer screening [24], Guerra et al. used CSR as means of validating what participants had said in the earlier, general phase of the interview. In the colon cancer screening study [23], focus groups were conducted to rank the importance of the barriers that had emerged using CSR in the qualitative interviews. Dee et al. [27] triangulated observation on the educational resources available in each practice with the findings on interviewees’ educational needs reported in CSR. However, the differential contribution of each method to the overall study results was not discussed.

Jennett et al. [29] compared the findings from CSR with those of chart audit. They found discrepancies between the two assessments; for instance, the impact of “several patient and physician characteristics, practice or professional factors, healthcare system and social factors...only became apparent through CSR”.

#### The role of the researcher: Training and reflexivity

Researchers with clinical backgrounds (see Table 3) conducted most interviews. Interviewer training was poorly described in most published reports, except in Lockyer et al. [28] which stated that the interviewer undertook training and pilots to ensure consistency of approach. Rochefort et al. [26] and Guerra et al. [23, 24] referred to the interviewer as “an evaluator that probes reasoning”, while Ab et al. [25] reported them as “non-confrontational”, but none of these studies discussed the impact of the interviewer on the interviewee any further. The authors of one study [13] reflected on the risk of medical interviewers introducing professional biases into the interview, and the measures taken to reduce this risk (i.e., involvement of non-clinical data coders).

#### Ethical concerns

Most studies, especially the more recent ones, reported ethical approval. However, few studies discussed ethical concerns specific to CSR. Ab et al. [25] addressed the issue of patient consent while Guerra et al. [23, 24] emphasized that no potentially identifiable patient information was required by the researcher, thereby protecting patient confidentiality.

### What type of findings might you expect?

CSR highlighted why certain actions were taken or not taken in chosen cases. For instance, the unrecorded social and clinical factors associated with **failure to implement guidelines** emerged in seven of the studies [13, 23–26, 28, 29]. These factors included the influence of unrecorded clinical signs and symptoms, antecedent knowledge of the patient [23, 24], patient demand [28, 29], and physicians’ personal opinions on guidelines [25, 26].

CSR demonstrated the **uncertainties** that occur for health care professionals in daily practice [25, 27], and physicians’ preferred resources to address these uncertainties [27, 28].

Studies using triangulation showed the **discrepancies between real and perceived behaviour** [23, 24, 29]. For instance, barriers reported by physicians in the opening phase of the qualitative interviews were often not apparent as barriers in the case data. Other barriers (such as physician forgetfulness) only became apparent during CSR.

CSR demonstrated **passivity in the provision of care.** Passivity can be difficult to capture in conventional qualitative interviews, as physicians may be blind to it. This was observed in the study on patients with multiple long-term conditions [13], where it emerged that physicians preferred to maintain the status quo in these patients rather than actively change their medications.

CSR facilitated exploration of the **less objective aspects of care** (e.g., assessments of life expectancy or patient preference) and the assumptions or knowledge on which these assessments [23–25] were based. The **influence of longitudinal care** can be shown by tracking decision-making over multiple consultations.

Referring to the chart helped ensure that low-priority issues were not overlooked in case discussions. For example, in Dee et al. [27] the uncertainty that arose in consultations may have been forgotten by participants had the chart not cued their recall. In some studies, CSR had an **educational ‘side-effect’**, by highlighting gaps in knowledge or deficiencies in care that the interviewee had previously been unaware of [23, 24, 28].

### Potential pitfalls and how to avoid them

#### Recall bias

While the purpose of CSR is to mitigate poor recall, Jennett et al. [29] demonstrated that using CSR alone remained prone to reporting biases. Chief among these was inaccurate post-hoc rationalisation. Physicians’ memories of clinical encounters are rarely complete, leading them to articulate “something having been done that really had not” [29]. As the physician may not accurately remember what they were thinking when making a decision, they retrospectively come up with explanations that make sense. This is compounded by the effect of social desirability on the interviewee, particularly if the interviewer is another healthcare professional. While Jennett et al. suggested that using chart audit in addition to CSR may reduce these biases, this is only useful if detailed and accurate data is available in the chart.

A shorter interval between the index consultation and the interview may facilitate recall. In Lockyer et al. [28], Dee et al. [27], and Sinnott et al. [13] the charts related to patients seen within two days of the interview. Guerra et al. [24] used charts for patients seen within the previous two weeks – even with this relatively short interval, multiple charts had to be excluded from the interviews as participants could not recall whether screening had been discussed with the patient. Rochefort et al. [26] used charts within the preceding year while Ab et al. [25] did not specify a time limit – neither study discussed the impact this had on recall.

#### Selection bias

It is possible that better record keepers are more likely to participate in studies that require access to medical records. As good record keeping is an indicator of quality in practice, studies using CSR risk recruiting a biased sample. For example, in Lockyer et al. [28] only those who prescribed phototherapy for jaundiced infants (some in accordance with guidelines and some not) were interviewed. The physicians who desisted from prescribing phototherapy (whether in accordance with guidelines or not) were not sampled. To counter such sampling effects, Rochefort et al. [26] used information from electronic medical records to stratify interviewees into those that rarely or mostly adhered to prescribing guidelines, and selected a maximum variation sample of cases for those participants.

#### Observation bias

Information provided by interviewees may be artefacts of the study itself. For example, in Dee et al. [27] it was not clear if the reported clinical uncertainties actually interfered with clinical care, or if they only arose as a product of reflection during CSR. None of the findings in Rochefort et al. [26] were related back to the patients whose charts were discussed. In Lockyer et al. [28] it was unclear if interviewees answered questions based on their management of the incident case that triggered the interview, or if their answers were rhetorical. Keeping the interview and the data analysis focused on the case data where possible may lessen the risk of observer effects.

## Discussion

In this scoping review, we have described the clinical research studies in primary care that have used chart stimulated recall. We identified eight clinical studies that used CSR, most of which had an emphasis on guideline implementation and adherence. From our analysis, it appears that referring to charts during qualitative interviews generates additional information on the influences on decision-making. None of the included studies offered any theoretical explanations to support their use of CSR. We suggest that the encoding specificity principle of memory [30] provides theoretical reasoning for how accessing contextual information about a patient encounter improves clinicians’ memory of that encounter. The principle states that memory is improved when information available at encoding is also available at retrieval. For example, the encoding specificity principle would predict that recall for information about a clinical decision is better if participants are interviewed while accessing the notes they wrote at the time of clinical decision-making. This may explain why study authors used CSR to gain greater specificity of detail, and explore not just what participants do but when they do it and why. Another explanation is that CSR may circumvent the difficulties GPs have in talking about diseases separately from the people who ‘have’ the disease [18]. Despite these utilities, we found that CSR was used in an inconsistent way and remains prone to biases that threaten its validity. CSR brings interviews closer to the reality of practice, but it does not completely close the gap.

### Strengths and limitations

The strengths of our review include a systematic search of the literature, which was augmented with manual searches of reference lists of published papers and systematic reviews. CSR is not a MeSH term so we used free text searches and a broad range of databases to capture relevant papers. A second strength is the quality assessment; although this is not usually a component of scoping review, it helped illuminate the strengths and weaknesses of included papers. Third, to facilitate interpretation of our findings and the use of CSR by other researchers, we analysed and reported our review using an established framework for the reporting of qualitative research techniques. A limitation of our review is the relative paucity of studies. The lack of a consistent definition of CSR increased the likelihood that we overlooked some papers. During the search, we found study protocols that outlined the intended use of chart-stimulated recall to evaluate the impact of knowledge transfer interventions on physicians’ behaviour [31]; once these results are available, they will add to the findings of our review. We restricted our analysis to the published accounts of the included studies for pragmatic reasons; greater justification for the approaches used may have occurred if authors were not restricted by word limits. Lastly, we were not able to determine the added value of using CSR in qualitative interviews in most studies.

### Unanswered questions and areas of future research to advance this method

#### Reflexivity and researcher training

In the assessment of professional competence, CSR interviewers are experienced clinicians who follow a six-month training programme on competence assessment [1]. However, the training of interviewers using CSR for clinical research has not been well described. To probe clinical reasoning, it may be advantageous for interviewers to have a clinical background, as doctors have been observed to give richer interviews with clinical researchers [32]. However, clinical researchers can change the dynamic of an interview if perceived by the interviewee to be a judge, a source of reassurance, or an expert [32]. Therefore, reflection on the impact of the clinical interviewer is required at the interview and the analysis stage, using input from a multidisciplinary research team [33]. None of the included papers discussed CSR training for non-clinical interviewers; this is another area which merits further evaluation and description. Empirical evaluation of the number of charts required to deliver optimal validity and the generalisability of CSR findings is also needed.

#### Ethics

There are ethical issues that are of specific concern in CSR but were not discussed in any of the published accounts. For example, unprofessional care or signs of physician burn-out may be identified in the interviews, and provisions should be made within the ethics protocol to deal with this scenario. Patient consent was rarely discussed. Researcher access to the charts or patient identifying information is not always necessary in CSR, but where it is necessary, patient consent would now be a mandatory requirement.

#### Other potential applications of CSR

CSR has been used in competence assessment for physical therapists [34] and occupational therapists [35], so it may have potential in clinical research in these specialities. We did not find evidence of its application in the assessment of decision-making in secondary care or by multidisciplinary care teams. Although it may be potentially useful in these settings, the technique would likely require modification to assess comprehensively the additional dimensions involved in team-based decision-making.

### Alternative approaches to CSR

CSR resembles other research techniques that use verbalisation to explore decision-making, each of which has its own strengths and weaknesses. An overview of these techniques and others used to stimulate concurrent or retrospective verbal reports in qualitative health research interviews is provided in Table 4. For example, in the think-aloud technique [36], participants verbalise all thoughts that come into their mind while actually performing a decision task. As a real-time approach, this may give more valid information than retrospective CSR. However, in acute clinical settings, it can slow down the decision process, is time-consuming and intrusive for participants, and can interfere with thinking [36, 37]. Case vignettes are a safe approach to explore decision-making but difficulties arise in determining the relationship between beliefs in a hypothetical situation and actions in real practice [38]. The critical incident technique involves participants discussing ‘bad’ or ‘good’ experiences in a specific area, thus can give skewed examples of care [39].

**Table 4:** Overview of a selection of techniques used to stimulate concurrent or retrospective verbal reports in qualitative health research interviews

	Description	Advantages	Limitations
Think-aloud technique [36]	Participants speak aloud any words in their mind as they complete a task or solving a problem. Can incorporate direct observation, audio or video recording.	As a real-time approach, may give more valid information than retrospective CSR. Links the thinking processes of the participant with concurrent perceptions, thus revealing information in working memory. May be combined with vignettes or simulations. May be used to study individual differences in performing the same task.	In clinical settings, is time-consuming and intrusive for participants. Given the limited capacity of memory, can hinder the participant’s cognitive processes, thus altering performance if tasks involve a high-cognitive load. Needs sufficient instructions and prompts from researcher to ensure sufficient verbalisation takes place, but prompts may then change how people think.
Case vignettes [38]	A brief carefully written description (or video/audio/photograph etc.) of a person or situation, designed to simulate key features of a real world scenario, is used to pose questions to a research participant.	Inexpensive, practical and safe approach to explore decision-making. Allows standardisation of cases across participants. The context or variables in the vignette can be varied across or within participants to address questions of interest.	Concerns that the artificiality of vignettes threatens external validity. Cannot test the relationship between beliefs in a hypothetical situation and actions in real practice. Risk of observer biases.
The critical incident technique [39]	Focuses on respondents’ accounts of events that have actually happened (incidents). Incidents are deemed to be critical when the purpose of the action and the outcome of the incident are reasonably clear and relevant to the phenomenon under study.	Systematic five-step process suited to the exploration of dilemmas. Encourages the natural tendency of people to tell anecdotes, but increases their value as data by focusing them onto a limited area of interest. Facilitates exploration of two sides of behaviour: good and bad; effective and ineffective; avoidable and unavoidable etc. Interview focuses on the specific reasons for actions and behaviours.	Focuses on skewed examples of care rather than routine cases. Operators may be reluctant to participate if they feel their performance is being scrutinized. Reliance on memory alone can result in data degradation and other data collection problems.
Video-stimulated recall [40]	A method of enhancing participants’ accounts of a consultation using a video-recording of the event to encourage and prompt recall in a post-consultation interview	A useful method to explore specific events within the consultation; mundane or routine occurrences; non-spoken events; and “taken for granted practice”. Visual stimuli may be a stronger stimulus for recall and allows participants to comment on non-verbal behaviours. Can be used to explore clinician and patient views.	Complex, costly and time-consuming. May generate a lot of data. Ethical hurdles of recording patient-encounters. Inappropriate probing during the interview may lead to reflection and analysis rather than recall of events as they happened.
Audio-stimulated recall [41]	A method of enhancing participants’ accounts of the consultation using an audio-recording of the event to encourage and prompt recall in a post-consultation interview	Facilitates analysis of conversation and verbal communication techniques. Less intrusive than video-recordings. Can be used to explore clinician and patient views.	Audios reproduce only a portion of the original experience. Inappropriate probing during the interview may lead to reflection and analysis of actions rather than recall of un-consciously produced patterns of action. Time consuming.
Chart- stimulated recall	During the interview, a patient’s chart is used as an aide-memoire to the physician’s recall of a case while the interviewer probes the reasons why certain decisions were made	Can add to the specificity of interview data, bridge the gap between real and perceived practice, and facilitate a deeper exploration of cognitive reasoning	Lack of information on use of chart-stimulated recall by non-clinical researchers, specific ethical issues and the number of charts required to give adequate representation of practice.
Protocol analysis [36]	A verbal process which reveals the “step-by-step” progression of a person’s problem-solving ability	Can reveal in detail the information that participants are concentrating on while performing their tasks. Can be combined with think-aloud technique.	Can be problematic in ill-structured tasks in complex environments. Has been criticized for being too reductive.
Material probes [42]	Objects (e.g. keepsakes, awards, trophies, and collectibles) or places (buildings, wards, open spaces) are used to prompt participants and elicit responses or memories during interviews.	Can help to keep the participant focused on a topic and provide a trigger for memories that might otherwise remain buried or actively excluded. Researcher can suggest materials or let participant choose them; participants can choose items they see as important to the topic, thereby adding to the depth of the discussion.	Allowing participants to choose objects can lead to the researcher losing some control over the interview and what is discussed. Choice of materials should be aligned with the research aim to ensure coherence across interviews.
Photo elicitation [43]	Inserting a photograph into a research interview to expand sensory awareness, increase the reflexive process and allow researchers to glean insights that might not be accessible via verbal-only methods.	Photos can be provided by the researcher, the respondent, or both. Elicit longer and more comprehensive interviews by overcoming the fatigue and repetition of conventional interviews.	Ethical issues regarding consent of photographed individuals. Participant chosen photos may veer towards the positive rather than negative end of their experiences. May not lead to a deep commentary if photos do not pose anything extra-ordinary or represent only taken-for-granted aspects work or activities.

## Conclusion

The limited use of chart-stimulated recall in clinical research to date undersells its potential to explore clinical decision-making. It can add specificity to qualitative interview data, bridge the gap between real and perceived practice, and facilitate a deeper exploration of cognitive reasoning. However, although CSR reduces some biases, it introduces others and a number of challenges lie ahead if CSR is to be adopted on a wider scale.

## Additional files

Additional file 1: Example search terms. Description of data: Terms used for scoping search of seven databases on the EBSCO platform. (PDF 287 kb)

Additional file 2: Data extraction form. Description of data: Data extraction form. (PDF 261 kb)

Additional file 3: PRISMA checklist. Description of data: PRISMA checklist. (PDF 441 kb)

Additional file 4: Quality appraisal of included studies. Description of data: Assessment of included studies using the CASP quality appraisal tool. (PDF 436 kb)

## Abbreviations

CSR: Chart stimulated recall
