# Supplementary material for: A scoping review of the potential for chart stimulated recall as a clinical research method
Source: BMC Health Serv Res. 2017 Aug 22;17:583. doi: 10.1186/s12913-017-2539-y (PMC5567630; doi:10.1186/s12913-017-2539-y)
Supplement: Supplementary file 1 — Example search terms. Description of data: Terms used for scoping search of seven databases on the EBSCO platform. (PDF 287 kb) [file 12913_2017_2539_MOESM1_ESM.pdf]

Additional file 1. Example search terms

Example of search terms used in CINAHL, via the EBSCOhost platfrom

#	Query	Limiters/Expanders	Last Run Via
**S1**	TX chart-stimulated recall OR TX chart stimulated recall OR TX case-based discussion OR TX case-based oral	Search modes - Boolean/Phrase	Interface - EBSCOhost Research Databases<br>Search Screen - Advanced Search<br>Database - CINAHL Plus with Full Text
**S2**	TX general practice AND TX general practitioner AND TX family practice AND TX family physician AND TX family medicine AND TX primary care AND TX primary health care AND TX primary medical care AND TX primary care physician AND TX family practitioner	Search modes - Boolean/Phrase	Interface - EBSCOhost Research Databases<br>Search Screen - Advanced Search<br>Database - CINAHL Plus with Full Text
**S3**	TX general practice OR TX general practitioner OR TX family practice OR TX family physician OR TX family medicine OR TX primary care OR TX primary health care OR TX primary medical care OR TX primary care physician OR TX family practitioner	Search modes - Boolean/Phrase	Interface - EBSCOhost Research Databases<br>Search Screen - Advanced Search<br>Database - CINAHL Plus with Full Text
**S4**	S1 AND S3	Search modes - Boolean/Phrase	Interface - EBSCOhost Research Databases<br>Search Screen - Advanced Search<br>Database - CINAHL Plus with Full Text
