# Supplementary material for: A scoping review of the potential for chart stimulated recall as a clinical research method
Source: BMC Health Serv Res. 2017 Aug 22;17:583. doi: 10.1186/s12913-017-2539-y (PMC5567630; doi:10.1186/s12913-017-2539-y)
Supplement: Supplementary file 2 — Data extraction form. Description of data: Data extraction form. (PDF 261 kb) [file 12913_2017_2539_MOESM2_ESM.pdf]

Additional file 2. Data Extraction Form

Author	
Year of publication	
Aims (Phenomena of Interest)	
Setting of study	
Participants: how sampled<br>• GPs (n)<br>• Others(profession, n)	
How were charts chosen	
How was topic guide/ interview schedule developed and used	
Methodology for data analysis	
Main findings	
How CSR was useful in generating these findings	
Duration of interviews	
Professional background and training of interviewer	
Other issues of note (esp. potential pitfalls and how to avoid them)	
