# Supplementary material for: A scoping review of the potential for chart stimulated recall as a clinical research method
Source: BMC Health Serv Res. 2017 Aug 22;17:583. doi: 10.1186/s12913-017-2539-y (PMC5567630; doi:10.1186/s12913-017-2539-y)
Supplement: Supplementary file 4 — Quality appraisal of included studies. Description of data: Assessment of included studies using the CASP quality appraisal tool. (PDF 436 kb) [file 12913_2017_2539_MOESM4_ESM.pdf]

Additional file 4: Critical Appraisal Skills Programme (CASP) tool for qualitative research

	Ab [28][i]	Dee [29]	Guerra [25]	Guerra [24]	Lockyer [27]	Jennett [23]	Rochefort [26]	Sinnott [13]
1 Was there a clear statement of the aims of the research?	y	y	y	y	y	y	y	y
2 Is a qualitative methodology appropriate?	y	y	y	y	y	y	y	y
Detailed Questions:								
3.Was the research design appropriate to address the aims of the research?	y	y	y	y	~[ii]	y	y	y
4.Was the recruitment strategy appropriate to the aims of the research?	y	y [iii.]	y	y	y	y	y	y
5. Were the data collected in a way that addressed the research issue?	y	~iv	y	y	~v	y	y	y
6 . Has the relationship between researcher and participants been adequately considered?[vi]	n	n	n	n	y	n	n	y
7 Have ethical issues been taken into consideration?								
-REC approval	y	n	y	y	n	n	y	y
-Ethical concerns[vii]	n	n	n	n	n	n	n	n
8 Was the data analysis sufficiently rigorous?	y	n[viii]	y	y	~[viii]	~[viii]	y	y
9 Is there a clear statement of findings?[ix]	y	y	y	y	y	y	y	y
10 How valuable is the research?	y	y	y	y	y	y	y	y

Notes:

[i] For consistency, the reference numbers for included studies in this file are the same umbers used in the main manuscript.
[ii] The interviews in this study used survey type questions – a more appropriate design would have included open ended questions. It was difficult to see what the charts added here (Lockyer) .
[iii] Sampling and recruitment for these studies was generally not easy: In Dee it took an "extensive search…considerable effort, patience and accommodation", 6% agreed in Jennett, 20% in Guerra, 36% Rochefort, 70% of those sampled in Ab (3 refused because of time constraints), 70% in Lockyer (others couldn't because on shift work), Sinnott –recruited by sign up at CPD – only one refused after initially saying yes (unpublished data).
[iv] Information in Dee was collected to show what clinical questions on reflection. They did not demonstrate if these questions interfered with care, or if the doctors would have actually gone on to seek answers to them. So the findings could have been an artefact of the study rather than a clinical reality.
[v] In Lockyer, it wasn't clear if the physicians answered questions based on the chart of the baby that led to the interview being triggered, or whether their answers to the Likert scale type questions were more rhetorical or free-floating.
[vi] Although the professional background of the researcher was given in Dee, Jennett, both Guerra studies, Lockyer, Sinnott; it was only the Sinnott paper where there was a discussion on how this may have impacted on the interviewee and on the data, and mentioned that the interview was guided by the participant as much as possible. Training of the interviewer was discussed in Lockyer. Ab said that interviews were non confrontational and open-ended questions used.
[vii] While most of the studies, especially the more recent ones described ethical approval, none discussed ethical concerns specific to CSR – for example, if the interviews reveal care that is concerning for suboptimal practice that places patients at risk, or if interviews show evidence of physician stress or burn out. Ab described patient consent to chart review, none of the others described patient consent. Both Guerra studies reported that no patient identifying information was required by the researcher, and in Sinnott no confidential patient info was accessed by or removed from the practice by the researcher (unpublished data)
[viii] Qualitative data analysis only briefly mentioned or not discussed.
[ix] The qualitative findings were not related back to the charts discussed in Rochefort (ie quotes do not concern cases) and there are no qualitative findings (ie quotes) in Dee or Lockyer. Overall sense that Dee were not conducted as a rigourous piece of qualitative research. Lockyer was evaluation of dissemination strategy using qualitative means.
